# Supplementary figures and images for: YAP inhibits HCMV replication by impairing STING-mediated nuclear transport of the viral genome
Source: PLoS Pathog. 2022 Dec 1;18(12):e1011007. doi: 10.1371/journal.ppat.1011007 (PMC9746980; doi:10.1371/journal.ppat.1011007)

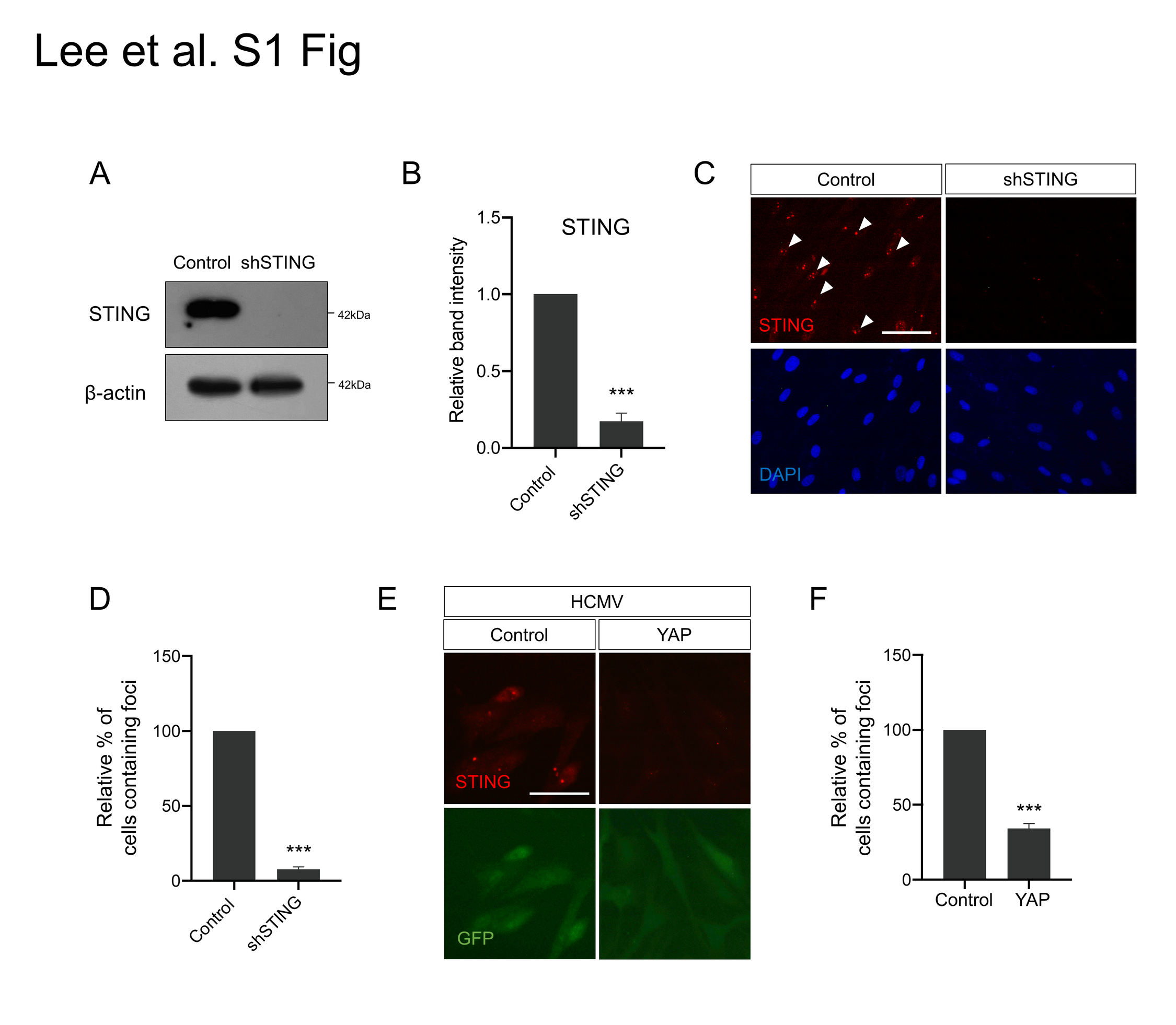

Supplement: S1 Fig — (A) Western blot and (C) immunocytochemical analyses of STING proteins using STING shRNA-transduced HFF cells at 2 days post-transduction. Closed arrow heads in (C) indicate STING foci. (E) YAP-transduced HFF cells infected with HCMV were immunostained using anti-STING antibody (red). (B, D, F) Quantification of band intensities and the number of STING+ foci in (A, C, E), respectively. Scale bars, 100 μm. n = 3 biological replicates for each experiment. Error bars represent SEM. Student’s t-test was used to determine statistical significance. ***P < 0.001. (TIF) [file ppat.1011007.s002.tif]

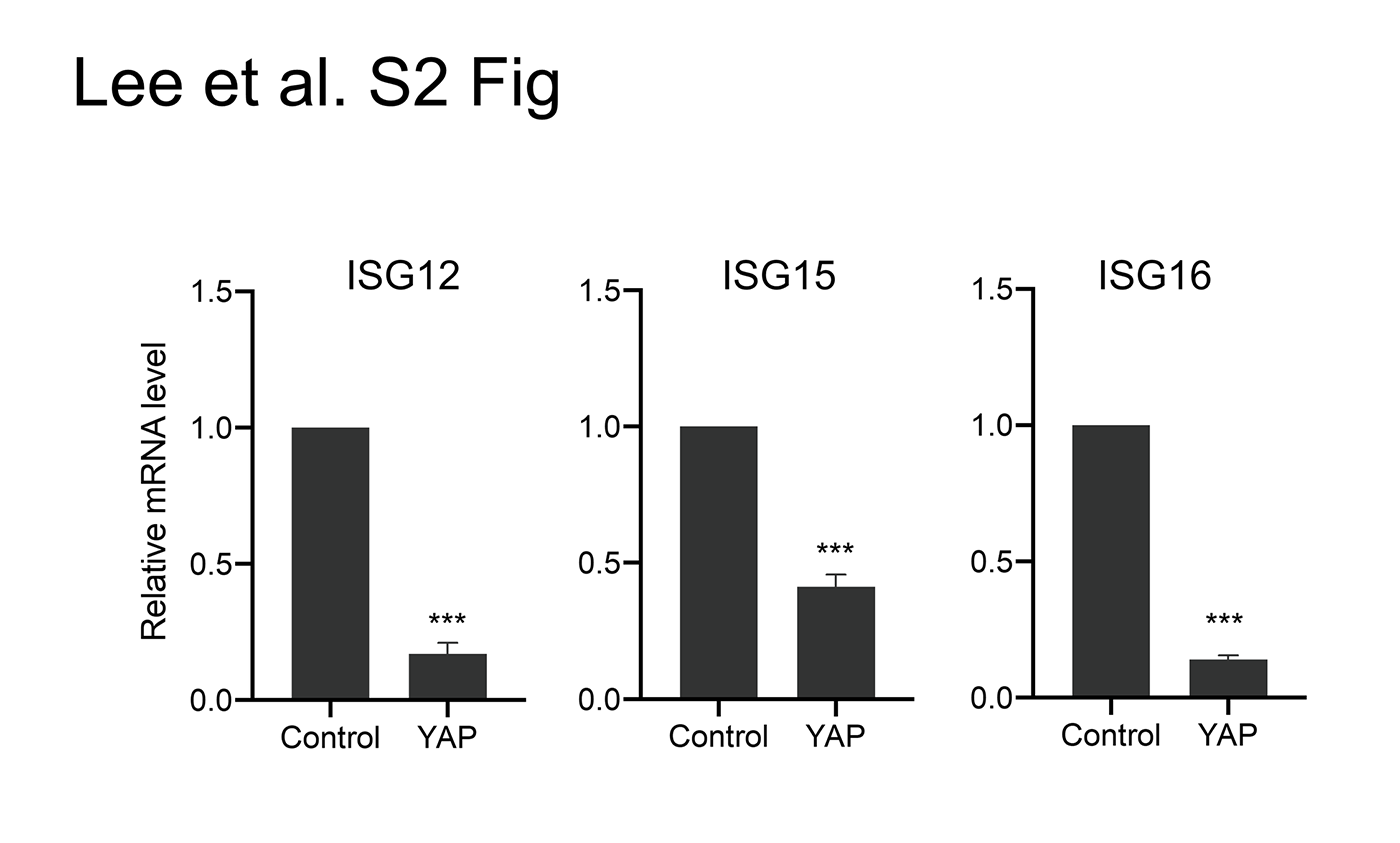

Supplement: S2 Fig — qPCR analysis of interferon-stimulated genes (ISGs) were performed using YAP-expressing HFF cells at 2 days post-transduciton. n = 3 biological replicates for each experiment. Error bars represent SEM. Student’s t-test was used to determine statistical significance. ***P < 0.001. (TIF) [file ppat.1011007.s003.tif]
