# Supplementary material for: YAP inhibits HCMV replication by impairing STING-mediated nuclear transport of the viral genome
Source: PLoS Pathog. 2022 Dec 1;18(12):e1011007. doi: 10.1371/journal.ppat.1011007 (PMC9746980; doi:10.1371/journal.ppat.1011007)

Lee et al. Fig 1G. Uncropped gel images

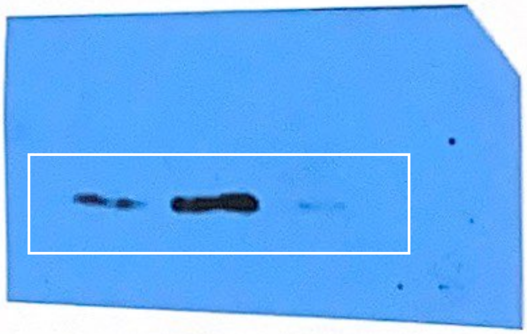

YAP

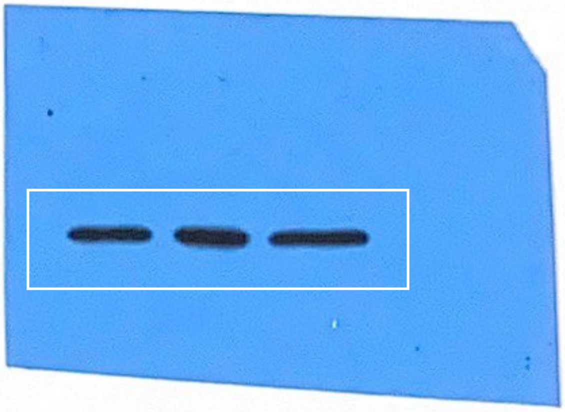

actin

Lee et al. Fig 2B. Uncropped gel images

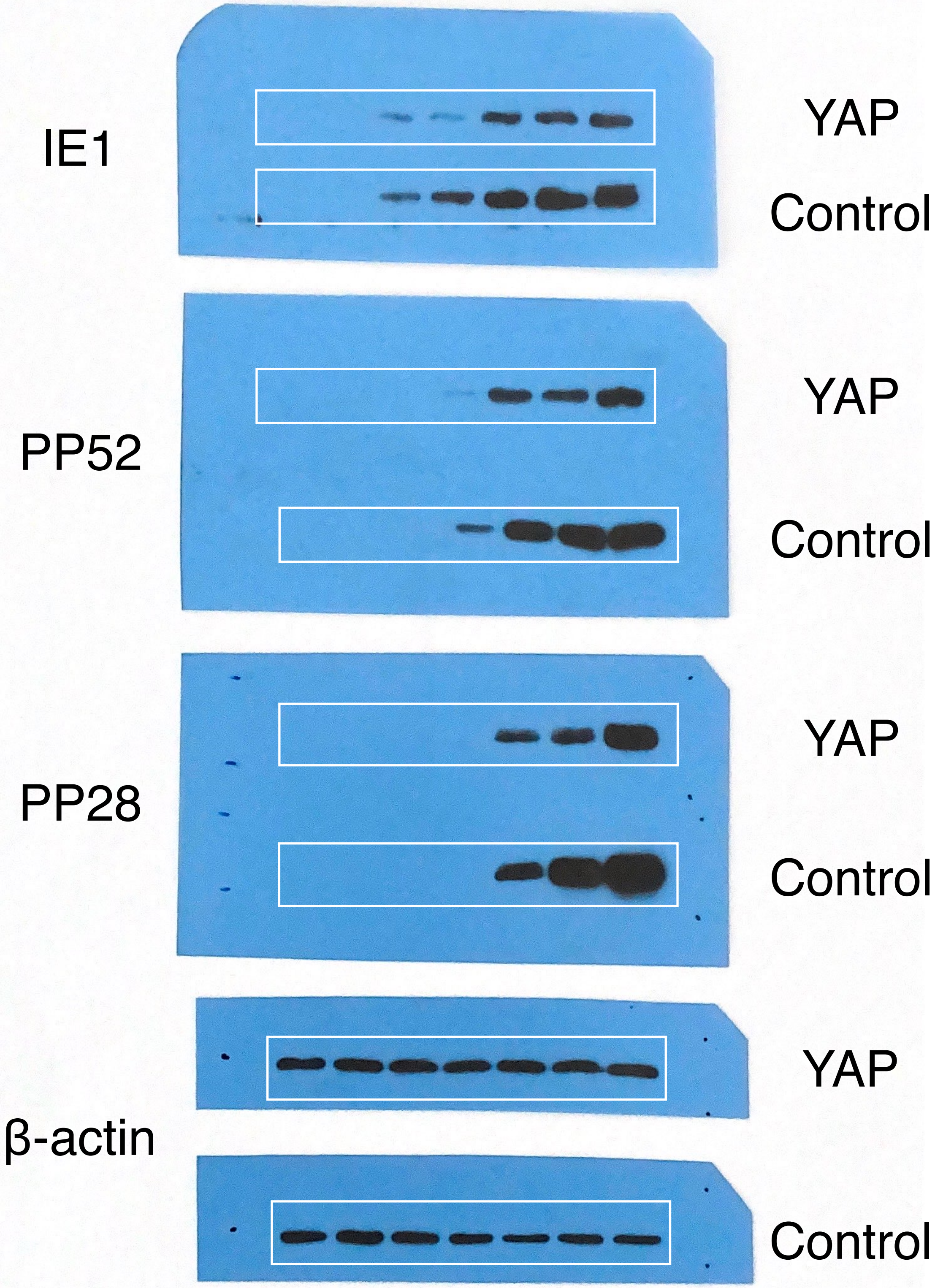

Lee et al. Fig 4C. Uncropped gel images

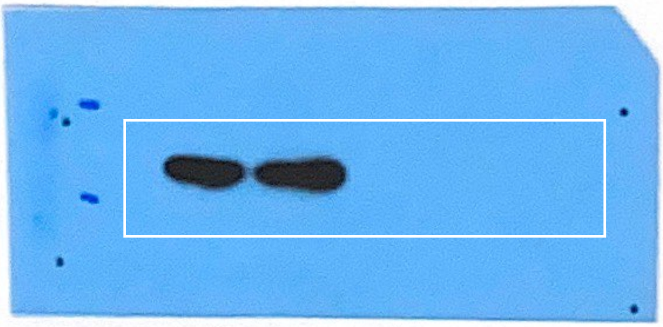

GAPDH

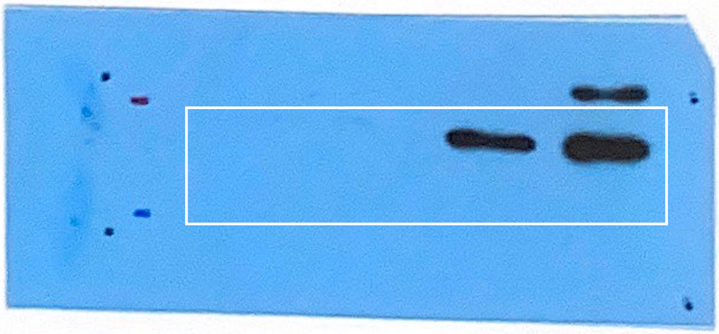

LAMIN A/C

Lee et al. Fig 4D. Uncropped gel images

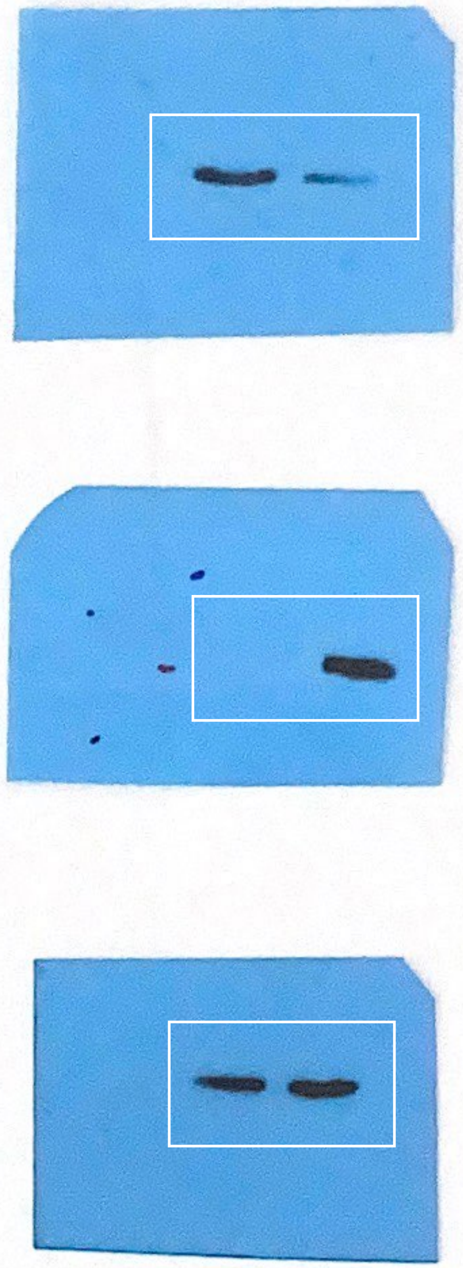

STING

YAP (myc)

actin

Lee et al. Fig 4N. Uncropped gel images

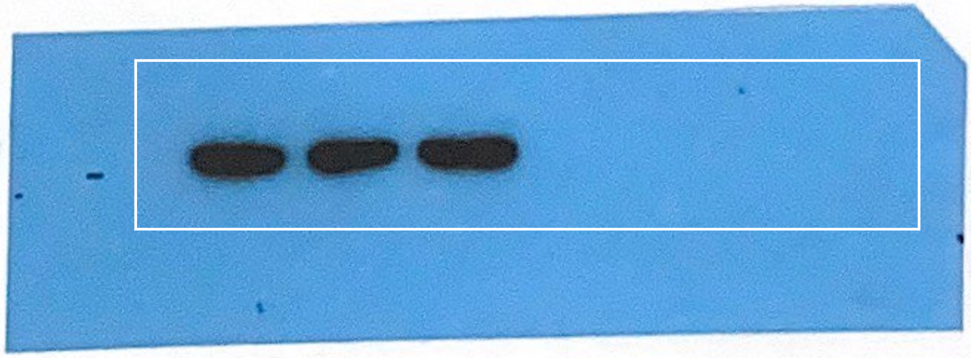

GAPDH

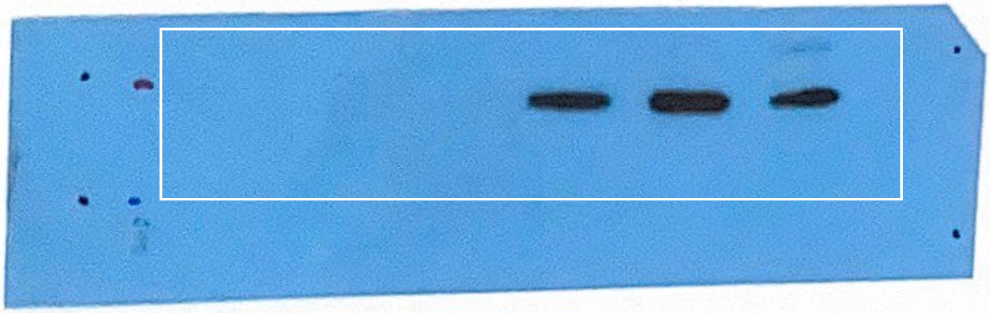

LAMIN A/C

Lee et al. Fig 4S. Uncropped gel images

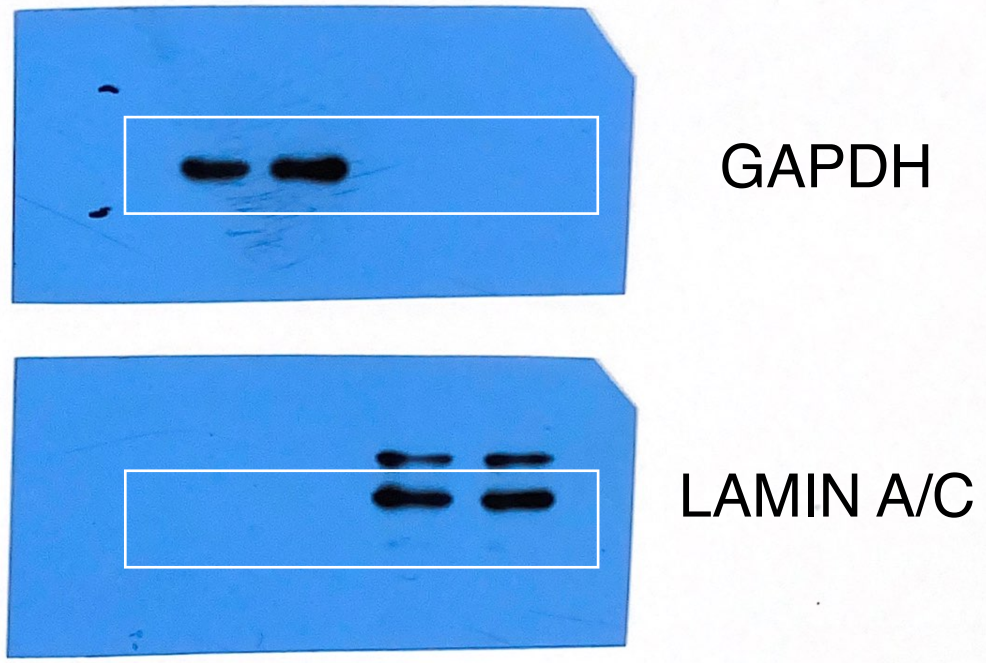

Lee et al. Fig 5C. Uncropped gel images

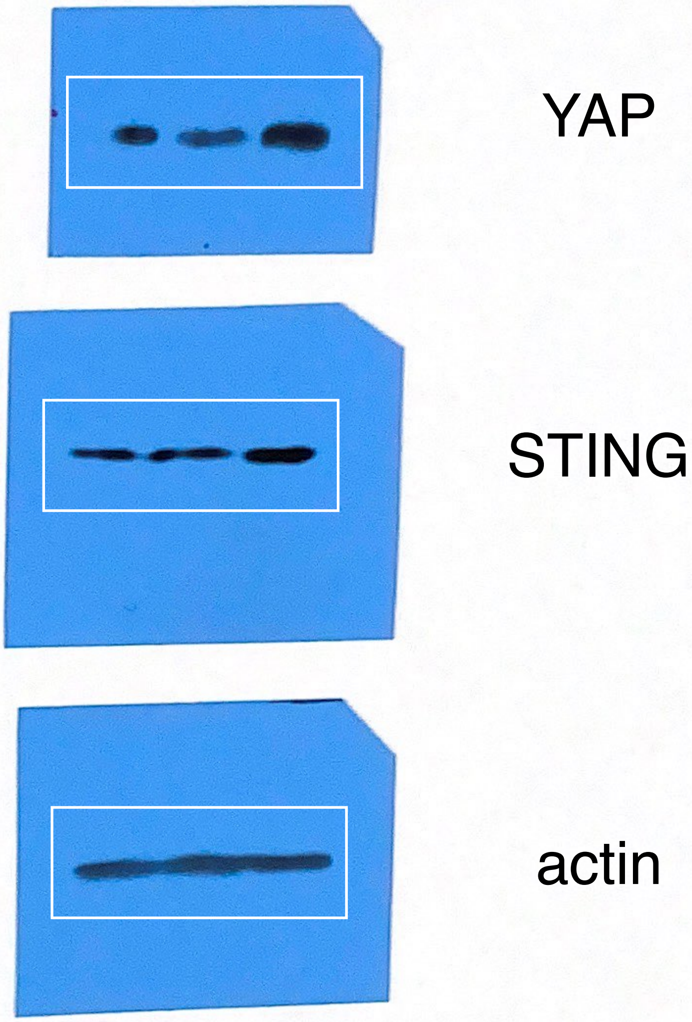

Lee et al. S1 Fig A. Uncropped gel images

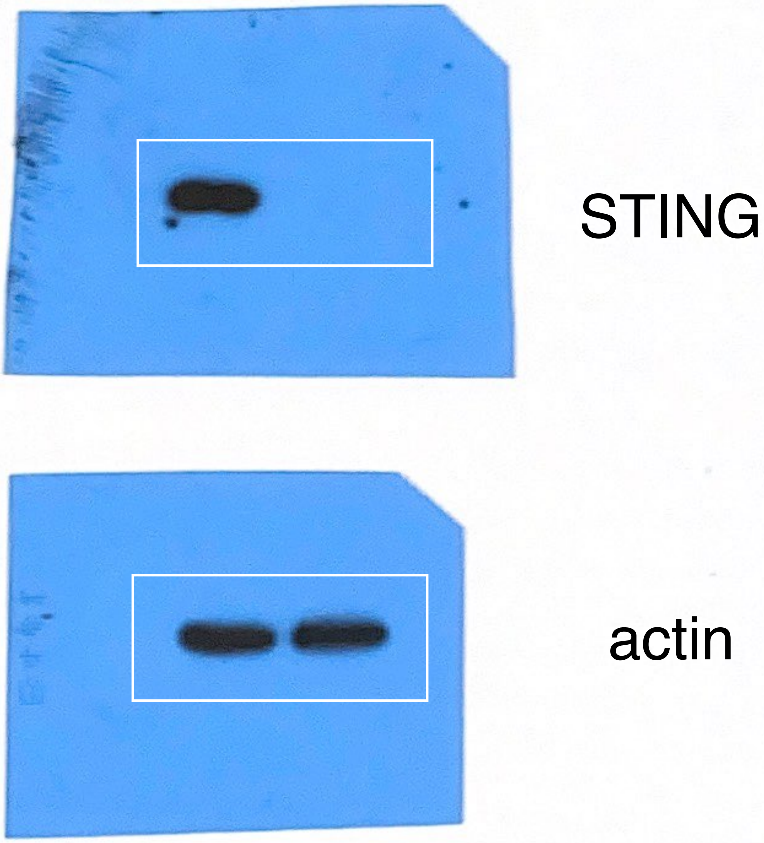

Supplement: S3 Fig — (PDF) [file ppat.1011007.s004.pdf]
