# Supplementary material for: YAP inhibits HCMV replication by impairing STING-mediated nuclear transport of the viral genome
Source: PLoS Pathog. 2022 Dec 1;18(12):e1011007. doi: 10.1371/journal.ppat.1011007 (PMC9746980; doi:10.1371/journal.ppat.1011007)
Supplement: Attachment — Submitted filename: 2022 10 28 HCMV-YAP Response to reviewers.docx [file ppat.1011007.s005.docx]

**Response to reviewers**

**Part I - Summary**
Please use this section to discuss strengths/weaknesses of study, novelty/significance, general execution and scholarship.

**Reviewer #1:**

In this paper, the authors revealed that YAP, well known for its involvement in the mammalian Hippo signaling pathway, inhibits HCMV replication by STING-mediated nuclear delivery of the viral genome. The authors observed that YAP inhibits the expression of STING at least at the transcription level, and showed that overexpression of STING is able to restore YAP-attenuated HCMV replication. STING is a well-known nucleic acid sensor that has long been known to activate the anti-viral interferon responses, and has recently been shown to be a pro-viral protein that promotes the nuclear delivery of herpesvirus genomes. Based on this previous report, the authors concluded that YAP inhibits the expression of STING, and consequently the nuclear transfer of HCMV genome is impaired, thereby inhibiting infection. It is a very interesting result, but the data is still premature to support the authors' conclusions. Further in-depth experiments are needed to reinforce the authors' conclusions. In particular, it is necessary to delineate what molecular mechanism YAP, the core subject of this paper, regulates the expression of STING.

**Reviewer #2:**

In this manuscript, Lee and colleagues examine the role of the Hippo signaling pathway and YAP transcription factor in viral infection, namely the b-herpesvirus HCMV. They find that YAP is an inhibitor of early HCMV life cycle, by inhibiting STING-dependent trafficking of the viral genome. In general, this manuscript details a new and potentially interesting link between Hippo signaling/YAP, STING expression and HCMV replication. However, further control and mechanistic experiments are required in order to fully support and strengthen the proposed hypothesis, including a physiological method of examining the effects of YAP on HCMV replication apart from over-expression. Further, the established role of STING in anti-viral cellular immunity and cell viability after viral infection must be addressed. Finally, controls confirming the validity of STING antibody staining are necessary.

Major Points
1. In Figure 1 over-expression experiments, what are the wt and over-expression levels of YAP in cells? Is the degree of YAP over-expression physiological, in terms of relation to wt levels? Does attenuation of endogenous YAP expression have a discernible effect on HCMV replication? The control vector should ideally express an inert protein, because ectopic protein expression in itself could inhibit viral replication.

> We thank you very much for your valuable comments and suggestions. We agree with the reviewer’s point that the amount of protein produced by a vector-mediated overexpression system would be higher than that at the physiological level. Before the YAP attenuation experiment, we screened for host cells that permit productive HCMV infection and also have a high level of basal YAP expression, and found no suitable cells satisfying both conditions. Under physiological conditions, YAP activation can be achieved by reduced activity of LATS1/2, a kinase directly upstream of YAP that induces YAP degradation. Then, we increased endogenous YAP expression using a dominant negative form of LATS1/2 (dnLATS1/2) (Fig 1G). As shown in Fig 1I and IJ, expression of dnLATS1/2 potently reduced HCMV progeny production, and coexpression of YAP shRNA vectors significantly increased progeny production. These data suggest that YAP-reduced HCMV replication can be recapitulated in physiological conditions.

> In addition, thanks to the insight obtained from the reviewers, we show that endogenous YAP increased by HCMV infection (Fig 5A and 5C) represses STING expression (Fig 5F).

> In the Fig 1 legend in the original manuscript, we indicated that the control vectors contain the GFP gene and that the YAP vectors express YAP and GFP bicistronically. To increase readability of the manuscript, we added an illustration to the Fig 1A in the revised manuscript showing the structure of the viral vector we used in this study for overexpression.

2. Does YAP over-expression alone adversely affect intrinsic cellular viability or replication, which would inhibit HCMV viral progeny? What about the YAP mutants?

> YAP is known as a positive, and not harmful, regulator of cell proliferation. According to the HCMV infection protocol, we prepare HFF cells and wait until the cell reach 100% confluence before infection. Therefore, additional cell proliferation is not likely to occur during HCMV infection.

> To test if YAP overexpression has adverse effects on cellular viability, we harvested samples 4 and 24 h after the transduced cells reached 100% confluence and performed the XTT assay. We chose 4 and 24 h considering the two reviewers’ similar comments (reviewer 1’s comment is in Part II – Major Issues). As shown in Fig 3B, overexpression of YAP and all its derivative mutant genes showed no toxic effect on cell viability.

3. Protein levels in Figure 2B (western blot) would be most useful if quantified.

> In the original manuscript, the protein levels in Figure 2B were quantified in Fig 2C.

4. Does YAP expression have an effect on levels of anti-viral type I or type III IFN cytokines, before or after expression?

> The reviewer’s comment has helped us study the impact of Yap on the cellular immune system in more depth. Since STING is a known key antiviral regulator that induces interferon (IFN) gene expression, it is important to test whether YAP-repressed STING expression would lead to subsequent changes in interferon expression. As shown in Fig 4K, YAP expression significantly reduced production of IFN-α and IFN-β (type I IFN), and IFN-λ (type III IFN). In addition, we found that interferon-stimulated genes (ISGs) were also downregulated by YAP (S2 Fig).

5. The authors state that STING typically localizes to the nucleus, however in unstimualted cells STING typically localizes to ER membrane. After stimulation with DNA or cyclic-di-nucleotides, STING typically re-localizes to ER-adjacent peri-nuclear structures, and co-localizes with TBK1. It is unclear what the structures in Figure 5E correspond to, as they appear to be detectable in unstimulated cells. Can the authors confirm these structures actually correspond to STING protein? If STING level are drastically different, why is the overall STING intensity not different comparing the control and YAP expressing cells in Figure 5E (e.g. staining outside the foci)?

> The foci structure formed by STING proteins was well described by the Vincent group (Gaston et al., 2016).

> To confirm whether the foci in Fig 4H actually correspond to STING proteins in our experimental settings, we performed immununostaining after shRNA-mediated STING knockdown. STING shRNA very effectively reduced STING protein levels (S1 Fig A and B) and the number of foci (S1 Fig C and D), confirming that the foci were indeed composed of STING proteins. In addition, STING shRNA also reduced immunostaining signals located outside of the foci.

> Even proteins which form special insoluble structures tend to exist in a soluble form at lower concentrations. Likewise, STING should form foci when a particular critical intracellular concentration is crossed. We inferred that YAP repression of STING gene expression was enough to prevent STING from reaching the high concentration required to form foci, but it was not strong enough to remove all residual soluble STING proteins in the cytoplasm, which is the STING staining outside the foci that the reviewer mentioned. qPCR and Western blot analyses showing that YAP significantly reduces STING expression but not as strongly as STING shRNA (Fig 4D-F vs. S1 Fig A and B) support this explanation.

* Reference

Gaston J, Cheradame L, Yvonnet V, Deas O, Poupon M-F, Judde J-G, Cairo S, Goffin V (2016) Intracellular STING inactivation sensitizes breast cancer cells to genotoxic agents. Oncotarget 7

6. Does STING directly associate with HCMV viral genome sequences in HFF?

> The Ahn group reported a tripartite interaction between STING, Nup358 (a component of the cytoplasmic filaments of the nuclear pore complex), and UL86 (the HCMV major capsid protein), and showed that this interaction promotes HCMV viral genome delivery to the nucleus (Hong et al., 2021). That is, STING directly associates with HCMV capsid proteins, and not the viral genome.

* Reference

Hong Y, Jeong H, Park K, Lee S, Shim JY, Kim H, Song Y, Park S, Park HY, Kim VN, et al. (2021) STING facilitates nuclear import of herpesvirus genome during infection. Proc Natl Acad Sci U S A 118: e2108631118

7. How do the authors rationalize lower STING levels (and presumably lower IFN levels) and lower HCMV replication? Does the effect of YAP over-expression change later in infection, due to paracrine effects of IFN?

> We appreciate the reviewer's comment that helped us think more deeply about the implications of our research.

> We understood the reviewer’s comment as a question about how our findings can be interpreted in terms of the overall HCMV life cycle. In the original and revised paper, we show that YAP-repressed STING expression results in impaired HCMV viral genome delivery and reduced IFN production. These findings imply that YAP prevents excessive multiple viral infection which would cause more damage to host cells or break latency, and that YAP enables HCMV to avoid immune attacks. Putting these together in terms of HCMV infection, we hypothesize that HCMV may use YAP to stably maintain the infection state by keeping a low profile without destroying the host cells while evading the host antiviral immune response. We included these explanations in the Discussion.

Minor Points
1. Not all of the data panels are properly discussed in the results section (e.g. Fig 1C and 1E)

> Thank you very much for the careful reading of our manuscript. As pointed out, we discussed all data panels in the Results section.

2. In Figure 1B and 1D, the differences between the panels (e.g. MOI) should be clearly indicated.

> As pointed out, we indicated MOI in Fig 1C and 1E in the revised paper.

**Reviewer #3:**

This paper by Lee et al. describes that YAP which is a critical regulator of the Hippo signaling pathway is able to negatively affect HCMV replication at the level of viral genome nuclear import. Essentially, the authors demonstrate that overexpresson of YAP inhibits HCMV replication. Further experiments show that this happens latest at the stage of immediate early gene expression and requires the transcriptional activation function of YAP. As an underlying mechanism it is shown that YAP downregulates STING and since STING (as shown in a previous publication) is required for the nuclear import of viral DNA, inhibition of the import of viral DNA into the nucleus accounts for the negative effect of YAP on viral replication.
This is a rather short paper, however, it is well structured and most results are convincing. One major criticism, however, relates to the fact that all results were obtained upon conditions of strong overexpression of YAP in primary human fibroblasts.
Thus, the major queston that needs to be answered by the authors is: does YAP indeed affect HCMV replication in the context of physiologically relevant conditions? The authors need to provide data concerning endogenous YAP levels to identify cell types that express high levels of YAP. Kockdown and infection experiments should then be used to show that YAP mediated inhibition of nuclear import is indeed a physiologically relevant mechanism that affects HCMV replication. Furthermore, it would be interesting to see whether YAP also affects other herpesviruses (e.g. herpes simplex virus).

**Part II – Major Issues: Key Experiments Required for Acceptance**

Please use this section to detail the key new experiments or modifications of existing experiments that should be absolutely required to validate study conclusions.

Generally, there should be no more than 3 such required experiments or major modifications for a "Major Revision" recommendation. If more than 3 experiments are necessary to validate the study conclusions, then you are encouraged to recommend "Reject".

**Reviewer #1:**

1. What are the dynamics of spatio-temporal expression of YAP and STING expression during HCMV infection?

Before responding to the reviewer’s two groups of comments,

> Many of the reviewer’s questions concern changes in endogenous YAP levels after HCMV infection.

> In this study, we report YAP-impaired HCMV nuclear transport. Since nuclear transport occurs long before viral gene expression and YAP can be activated by diverse cellular events, initially it did not seem logical to assess the alterations in YAP expression by HCMV infection. This is because not all biological events show positive or negative feedback interactions, and thus HCMV infection would not necessarily affect YAP expression.

> However, we did our best to answer the reviewer's questions. Now, we deeply appreciate the reviewer's comments because we were able to greatly extend the scope of our study from pre-HCMV infection to the post-infection stages by obtaining interesting and meaningful results. In addition, we were also able to incorporate the potential immunological role of STING into our research.

1) Authors should analyze the expression of YAP and STING during the immediate-early stage of HCMV infection.

> As suggested, we analyzed the expression patterns of YAP and STING after HCMV infection and found that overall YAP and STING expression were elevated during the early stages of the HCMV life cycle (Fig 5A-E). Enhanced STING expression is presumably attributable to a cellular event of host antiviral immune mechanisms, but the increase was less pronounced than that of YAP mRNA.

> Importantly, we show that HCMV-triggered STING mRNA expression can be further enhanced in the YAP knockdown conditions (Fig 5F). indicating that HCMV-induced YAP effectively minimizes the increase of STING expression. These data suggest that HCMV negatively regulates STING functions by increasing YAP. We added these data and explanation to the Results section in the revised paper.

HCMV infection appears to upregulate mRNA level of STING. What about changes in STING protein levels?

> Yes, HCMV infection increases the STING protein levels (Fig 5C and 5E).

Data show that overexpression of YAP inhibits the appearance of STING foci. Is a similar phenomenon observed in the context of HCMV infection?

> Yes, YAP also reduced the number of STING foci in the context of HCMV infection (S1 Fig E). We already showed that YAP-inhibited STING expression in the context of HCMV infection (Fig 4G) and verified that the foci was composed of STING proteins using TEAD shRNA (S1 Fig C). Therefore, the YAP-reduced appearance of STING foci in the HCMV-infected samples is not very surprising. Again, the mRNA-protein-foci of STING are tightly correlated with each other.

2) YAP protein is a core protein in Hippo signaling which regulates cell proliferation/death. Cell proliferation could mislead the quantification of the amount of DNA transferred into the nucleus. Therefore, it is important to rule out the possibility that the ectopic expression of YAP protein did not affect cell proliferation.

> Nuclear transport of the HCMV genome occurs at the earliest stage of the viral life cycle, and we harvest samples for subcellular fractionation at 4 hpi. 4 hr is too short a time for cell proliferation to occur.

> In addition, according to the experimental HCMV infection protocol, HCMV infection is carried out when host cell confluence reaches 100%. Therefore, additional cell number increase is unlikely to occur.

> However, to address the reviewer’s point experimentally, we performed the XTT assay measuring cell numbers under the same conditions used for HCMV infection up to 24 h and found almost no difference in the cell numbers between YAP and control samples (Fig 3B).

3) To clarify YAP represses HCMV viral gene expression through the regulation of STING expression, authors are encouraged to show that the Hippo signaling pathway is not activated during HCMV infection. It can be done by phosphor-immunoblotting for the components of Hippo signaling pathway components such as Hippo, MST1/2 and LATS1/2. Given the previous reports that the activity of YAP depends on its phosphorylation, it may provide valuable information to the direction of in-depth research to see if there is a correlation between the phosphorylation status of YAP and STING expression during productive HCMV infection?

> YAP activity can be regulated at both the transcriptional and post-translational levels. In this regard, the reviewer’s point is very appropriate.

> If the YAP mRNA levels had not changed, post-translational modification of YAP after HCMV infection would have been very important for YAP functions. However, Fig 5A shows greatly enhanced YAP mRNA expression upon HCMV infection, weakening the importance of post-translational regulation, although we cannot exclude the possibility of LATS1/2 regulation of YAP.

2. What are the molecular mechanisms by which YAP controls the cellular level of STING? Some specific questions are as follows.

1) Does HCMV manipulate the expression of STING through YAP related pathway?

> Yes, the reviewer’s insight was right. HCMV modulates STING expression through YAP.

> We have already provided an answer to the reviewer's similar comment 1 - 1) above.

2) Does HCMV infection affect the expression of endogenous YAP protein?

> Yes, HCMV infection increases the expression of endogenous YAP. Likewise, our detailed answer was explained in the response to the reviewer's comment 1 - 1) above.

> We thank the reviewer for the important comments. The reviewer’s suggestions made it possible to study the YAP-STING relationship in the context of HCMV infection.

3) Does YAP directly activate the STING promoter?

> Throughout this study, we show that YAP inhibits STING expression at the mRNA levels. Therefore, we interpreted the reviewer’s question as “Does YAP directly ‘suppress’ (instead of ‘activate’) the STING promoter?”. In the Discussion section of the original manuscript, we already provided possible direct or indirect mechanisms by which YAP might inhibit STING expression. During the revision period, we analyzed the STING promoter sequences and found no consensus TEAD binding sites in the promoter-proximal regions of STING gene. Therefore, it seems more likely that YAP suppresses STING expression indirectly, that is, by regulating transcription of an unknown activator or repressor for STING expression. We included this possibility in the Discussion section.

4) YAP functions as transcription factor by interacting with multiple co-activators such as TEAD family, Runx, and AP2. Although authors confirm that interaction between YAP and TEAD family is required for the function of YAP, it should be more thoroughly validated.

> This is an important question about the role of TEAD family transcription factors in YAP functions. To address this question directly, we performed a progeny assay using an shRNA-based TEAD knockdown approach. dnLATS1/2-upregulated endogenous YAP proteins (Fig 1G and 1H) (please see the response to the reviewer’s subsequent #5 comment below regarding the properties of dnLATS1/2) impaired HCMV progeny production and coexpression of TEAD shRNA completely abolished the effect of dnLATS1/2 (Fig 3G and 3H). In addition, qPCR results showing strong upregulation of STING mRNA levels by TEAD shRNA expression (Fig 4J) further supported the importance of TEAD in YAP functions. These data indicate that TEAD is critically required for YAP-inhibited HCMV replication and STING expression.

5) The gain-of-function study must be accompanied by a loss-of-function study to prove that it is not an artificial outcome. Overexpression of YAP decreased HCMV replication. Does depletion of YAP enhance HCMV replication? Try knockdown or knockout experiments of YAP and proceed to assay viral growth (RNA, protein level, and titration of progeny virus)

> We appreciate the reviewer’s point about the need of a loss-of-function approach. Host cells such as HFF used for experimental HCMV infection systems possess low levels of YAP proteins. To overcome this, we increased YAP proteins at the endogenous level by employing a dominant negative form of LATS1/2 (dnLATS1/2). LATS1/2 is an upstream kinase of YAP that facilitates YAP degradation. Then, we tested shRNA-mediated YAP knockdown. As shown in Fig 1G-1J, dnLATS1/2 increased YAP protein levels and greatly reduced HCMV progeny production. Cotransduction of YAP shRNA vectors significantly reverted dnLATS1/2-associated phenotypes. These approaches helped us investigate the effects of YAP expression and YAP depletion on HCMV replication under physiological conditions.

**Reviewer #2:**

We have already responded to the following reviewer’s comments in detail in the previous Part I-Summary section.

1. Endogenous YAP knockout or knockdown (loss of function) to validate over-expression results

> To test the effects of endogenous YAP activation and knockdown we used dnLATS1/2. Please refer to our response to the reviewer’s Major Point #1 in the Part I- Summary section.

2. Cell viability after YAP over-expression

> To test cell viability, we performed an XTT assay and found no significant difference in cell viability between YAP and control samples. We have provided a more detailed answer to the reviewer’s #2 comment above in the Part I-Summary section.

3. Validate STING protein staining for immunofluorescence experiments (Fig 5E)

> To validate if the spots in Fig 4H were composed of STING proteins, we performed immunofluorescence using STING shRNA-expressing samples. Please see our response to the reviewer’s Major Point #5 in the Part I-Summary section.

**Reviewer #3:**

1. The authors need to identify cell types and/or conditions where YAP is expressed at high levels. Knockdown experiments should then be performed in order to demonstrate that YAP indeed inhibits HCMV replication in a physiological context.

> The reviewer’s comment pointing out that our findings should be confirmed in a physiological context is important. As the reviewer suggested, we used a condition in which Yap is expressed at high levels within a range that does not exceed the physiological levels. Under physiological conditions, YAP activation can be achieved by reduced activity of LATS1/2, a direct YAP upstream kinase that induces YAP degradation. We increased endogenous YAP proteins using a dominant negative form of LATS1/2 (dnLATS1/2) (Fig. 1G and 1H). As shown in Fig 1I and 1J, we found that dnLATS1/2 expression potently reduced HCMV progeny production and coexpression of shRNA vectors against YAP restored progeny production. The dnLATS1/2-based approach helped us investigate the effects of YAP expression and YAP attenuation on HCMV replication at the physiological level, and yielded results consistent with the YAP overexpression experiments.

2. The authors should use other herpesviruses (e.g. HSV-1, VZV) as further controls in their experiments.

> We appreciate the reviewer’s very helpful comment. As the reviewer suggested, it is important to know whether the YAP-reduced STING expression can affect replication of other herpesviruses. To test this possibility, we chose HSV-1 based on a recent report* showing that HSV-1 capsid proteins bind to STING and that the efficient nuclear import of the HSV-1 genome is also STING-dependent (Hong et al., 2021). As shown in Fig 4R and 4S, we found that the nuclear transport of HSV-1 viral DNA was also significantly reduced by YAP expression. These results suggest that the nuclear delivery of any virus that requires STING can be inhibited by YAP activation. These data also greatly helped us to experimentally support the last sentence in the Discussion.

* Reference

Hong Y, Jeong H, Park K, Lee S, Shim JY, Kim H, Song Y, Park S, Park HY, Kim VN, et al. (2021) STING facilitates nuclear import of herpesvirus genome during infection. Proc Natl Acad Sci U S A 118: e2108631118

**Part III – Minor Issues: Editorial and Data Presentation Modifications**
Please use this section for editorial suggestions as well as relatively minor modifications of existing data that would enhance clarity.

**Reviewer #1:**

Several overlapping and redundant data with the same conclusion are presented in this manuscript. Figures 1 and 2 can be merged. Figures 4, 5, and 6 repeat the same information and must be merged together.

> As suggested, Figures 4, 5, and 6 were merged to Figure 4 in the revised paper. Figures 1 and 2 were left separate because the two figures have distinct aspects of viral replication, progeny generation and intracellular viral gene expression, and new data on the effects of endogenous YAP were added to Fig. 1.

**Reviewer #2:**

1. Not all of the data panels are properly discussed in the results section (e.g. Fig 1C and 1E)

> As pointed out, we discussed all of the data in the Results section.

**Reviewer #3:**

1. The authors need to indicate in each figure legend the number of biological replicates that was used to calculate the statistical significance.

> As commented, we indicated the number of biological replicates in each figure.
